# Supplementary material for: A Boronated Derivative of Temozolomide Showing Enhanced Efficacy in Boron Neutron Capture Therapy of Glioblastoma
Source: Cells. 2022 Mar 31;11(7):1173. doi: 10.3390/cells11071173 (PMC8998031; doi:10.3390/cells11071173)
Supplement: Supplementary file 1 [file cells-11-01173-s001.zip › Supplement Figures.pptx]

## Slide 1
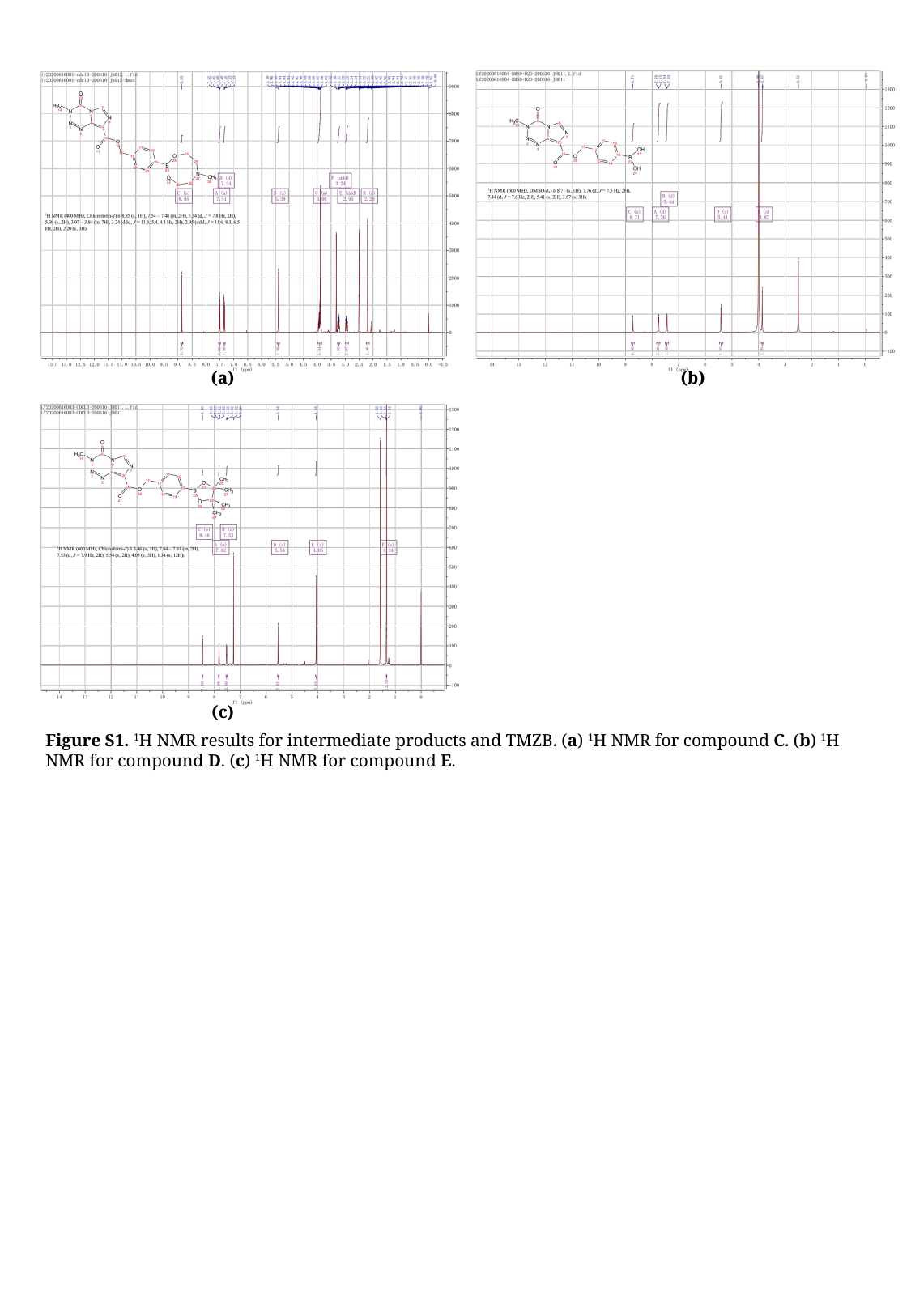

(b)
(a)
(c)
Figure S1. 1H NMR results for intermediate products and TMZB. (a) 1H NMR for compound C. (b) 1H NMR for compound D. (c) 1H NMR for compound E.

## Slide 2
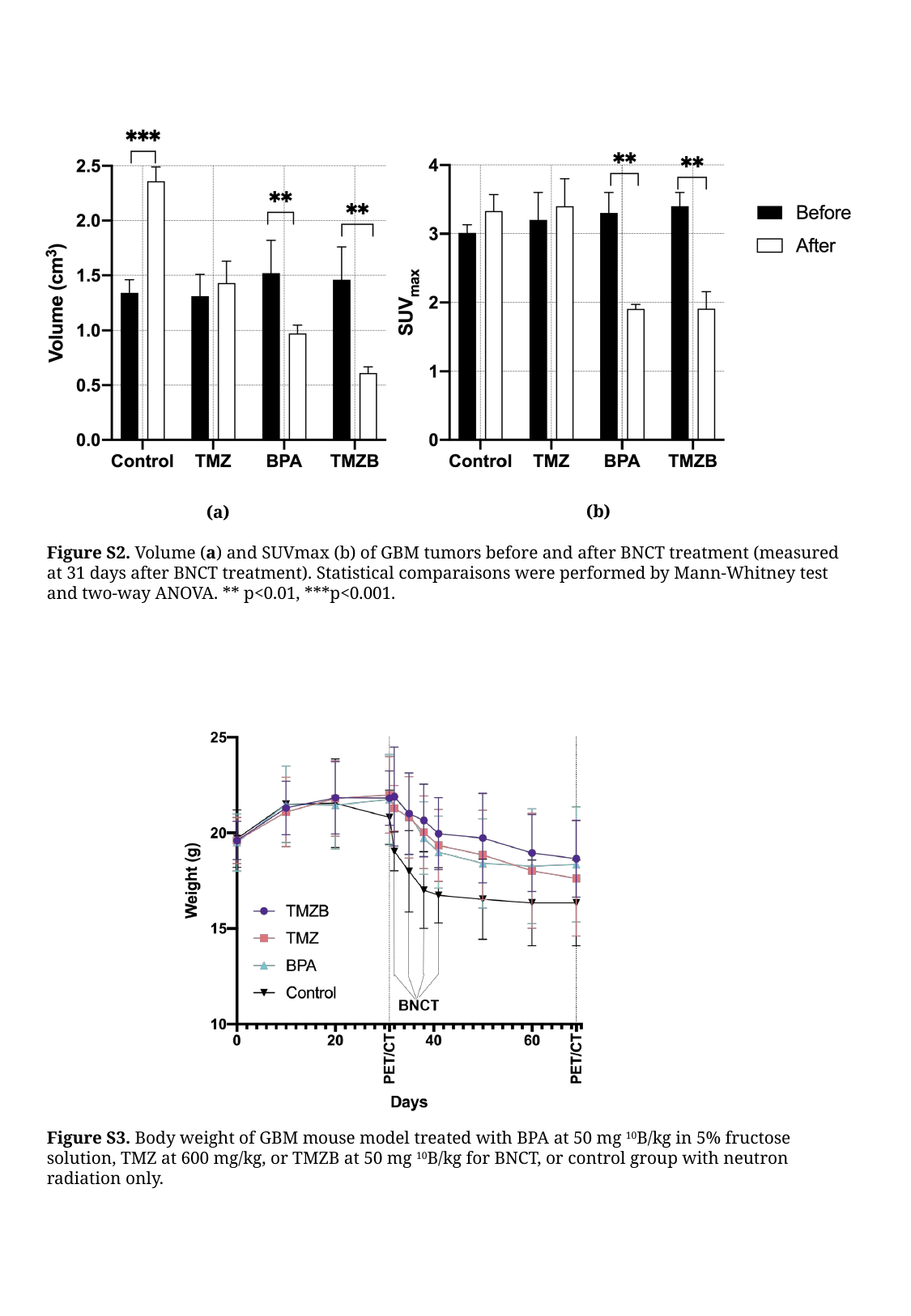

(b)
(a)
Figure S2. Volume (a) and SUVmax (b) of GBM tumors before and after BNCT treatment (measured at 31 days after BNCT treatment). Statistical comparaisons were performed by Mann-Whitney test and two-way ANOVA. ** p<0.01, ***p<0.001.
Figure S3. Body weight of GBM mouse model treated with BPA at 50 mg 10B/kg in 5% fructose solution, TMZ at 600 mg/kg, or TMZB at 50 mg 10B/kg for BNCT, or control group with neutron radiation only.
